# Zeta Potential Measurements on Solid Surfaces for *in Vitro* Biomaterials Testing: Surface Charge, Reactivity Upon Contact With Fluids and Protein Absorption

**DOI:** 10.3389/fbioe.2018.00060

**Published:** 2018-05-09

**Authors:** Sara Ferraris, Martina Cazzola, Veronica Peretti, Barbara Stella, Silvia Spriano

**Affiliations:** ^1^Department of Applied Science and Technology, Institute of Materials Physics and Engineering, Politecnico di Torino, Turin, Italy; ^2^Dipartimento di Scienza e Tecnologia del Farmaco, Università degli Studi di Torino, Turin, Italy

**Keywords:** zeta potential, biomaterials, *in vitro* testing, surface charge, reactivity, protein absorption

## Abstract

Surface properties of biomaterials (e.g., roughness, chemical composition, charge, wettability, and hydroxylation degree) are key features to understand and control the complex interface phenomena that happens upon contact with physiological fluids. Numerous physico-chemical techniques can be used in order to investigate in depth these crucial material features. Among them, zeta potential measurements are widely used for the characterization of colloidal suspensions, but actually poorly explored in the study of solid surfaces, even if they can give significant information about surface charge in function of pH and indirectly about surface functional groups and reactivity. The aim of the present research is application of zeta potential measurements of solid surfaces for the *in vitro* testing of biomaterials. In particular, bare and surface modified Ti6Al4V samples have been compared in order to evaluate their isoelectric points (IEPs), surface charge at physiological pH, *in vitro* bioactivity [in simulated body fluid (SBF)] and protein absorption. Zeta potential titration was demonstrated as a suitable technique for the surface characterization of surface treated Ti6Al4V substrates. Significant shift of the isoelectric point was recorded after a chemical surface treatment (because of the exposition of hydroxyl groups), SBF soaking (because of apatite precipitation IEP moves close to apatite one) and protein absorption (IEP moves close to protein ones). Moreover, the shape of the curve gives information about exposed functional groups (e.g., a plateau in the basic range appears due to the exposition of acidic OH groups and in the acidic range due to exposition of basic NH_2_ groups).

## Introduction

The biological response to implanted biomaterials strongly depends on their surface properties, such as roughness, chemical composition, charge, wettability and hydroxylation degree. These features drive the absorption of water molecules, ions and proteins, immediately after implantation and consequently affect the ability of materials to interact with cells and also with bacteria, eventually present at the implant site (Kasemo, 2002).

Numerous techniques can be employed for surface characterization of biomaterials (Voros et al., 2001): Field Emission Scanning Electron Microscopy (FESEM) is often used for morphological evaluation together with Atomic Force Microscopy (AFM). X-ray Photoelectron Spectroscopy (XPS) is applied for the investigation of chemical composition and chemical functional groups, as well as Fourier Transformed Infrared (FTIR) and Raman Spectroscopies. The same techniques can be employed for the investigation of protein absorption phenomena, together with ellipsometry, quartz crystal microbalance, circular dichroism, bicichroninic acid assay (BCA) and fluorescent o radiolabeling techniques (Tengvall, 2001; Rabe et al., 2011; Silva-Bermudez and Rodil, 2013).

Among them, zeta potential measurements are widely applied in the field of colloidal suspensions (for the investigation of their stability, Bhattacharjee, 2016), but they are still poorly considered in the case of solid surfaces and almost unexplored for the investigation of protein absorption on biomaterials. To this regard, zeta potential describes the surface charging behavior in contact with water based electrolytes and gives information about the isoelectric point, the surface charge in function of pH, but also (indirectly) of the functional groups exposed at the solid-liquid interface, the reactivity of the surface in the test solution and absorption processes (Luxbacher, 2014).

When a material comes into contact with water-based media (such as physiological fluids) a surface charge is developed at the interface. This phenomenon has different causes depending on the surface characteristics: in case of hydrophobic surfaces with no functional groups, the exposed surface charge is due to replacement of the adsorbed water molecules with ions (OH^−^, H_3_O^+^); on the other hand, in case of specific functional groups exposed on the surface, acid-basic reactions between the liquid medium and these groups can take part (e.g., dissociation of the hydroxyl groups or protonation of the amine groups) with consequent development of charges (Luxbacher, 2014).

**Figure 1:**
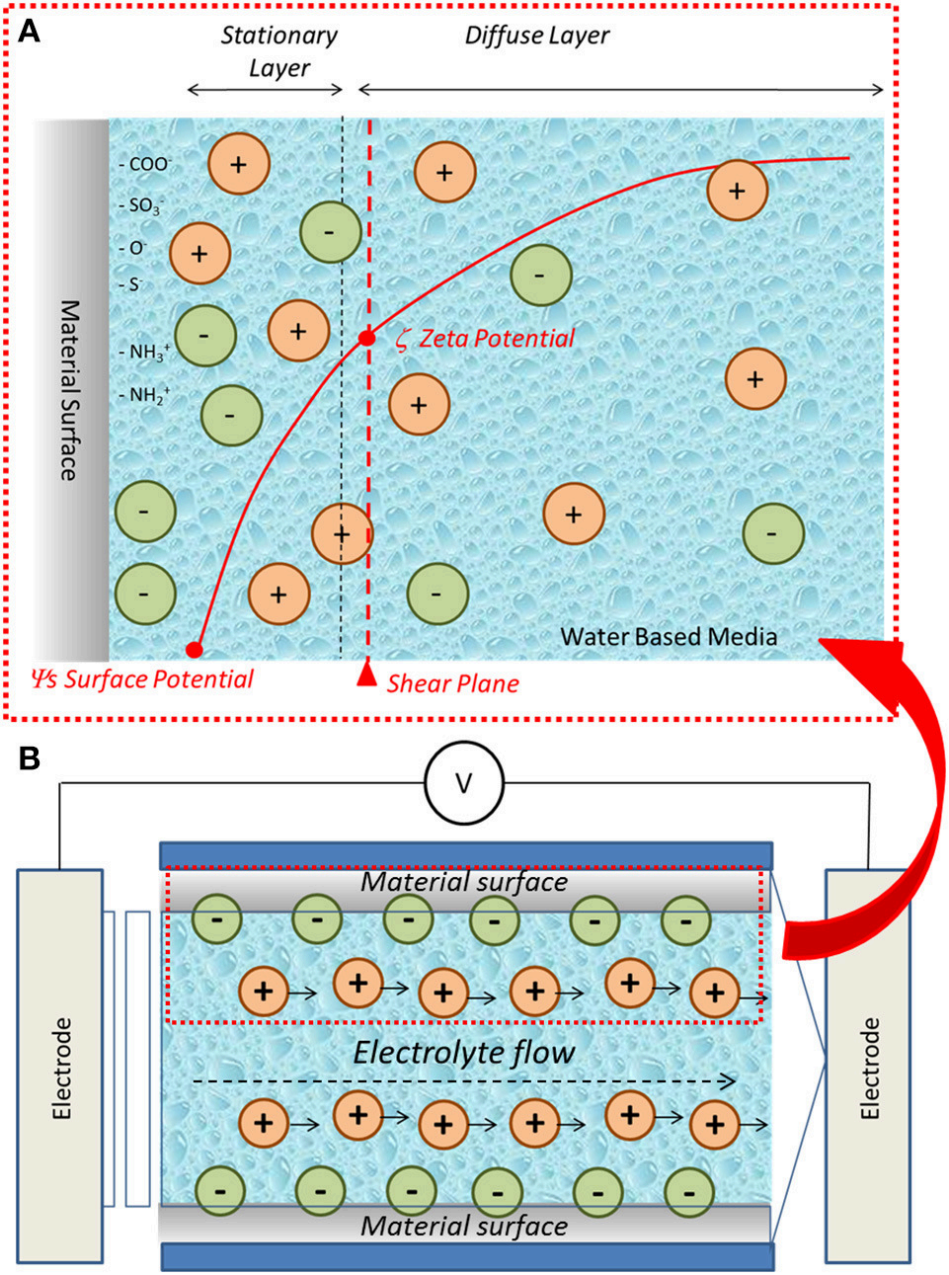
**(A)** Schematic representation of charge development at biomaterial-water based medium interface and zeta potential, **(B)** schematic representation of the streaming potential technique for the measurement of solid surface zeta potential.

The development of a charge at the solid-liquid interface is consequent to counterions distribution in the liquid. The surface potential decays increasing the distance from the surface. This phenomenon can be described by means of the Electrochemical Double Layer (EDL) model which defines a “*stationary layer*” (stationary immobile layer) and a “*diffuse layer*” (diffuse mobile layer) of counterions that compensate the surface charge. The zeta potential is defined as the potential at the outside of the stationary layer (Luxbacher, 2014). A schematization of the surface charge development at the solid-liquid interface and of the EDL model as well as of the surface and zeta potential definitions is reported in Figure 1A.

Considering the solid-liquid interface with the above described development of a surface charge, if the water based medium moves, its flow will cause the motion of the counterions, that compensate the surface charge, in the direction of the flow itself. The consequent charge separation causes a difference in the electrical potential called streaming potential. This physical entity can be used for the determination of the zeta potential of solid surfaces by means of the so called streaming potential technique (Luxbacher, 2014) (Figure 1B). This method has been employed in the present research for the characterization of bulk biomaterials.

In particular, the study of different surface modifications, of *in vitro* bioactivity and of protein absorption have been carried out in the present research by means of zeta potential titrations on solid surfaces. At the best of author knowledge, this is the first time in which this technique is employed for the investigation of surface modifications, bioactivity and protein absorption. The obtained results suggest that zeta potential measurements can be extremely sensitive and useful in the in depth understanding of surface properties of biomaterials.

## Materials and methods

### Samples preparation

Ti6Al4V disks (2 mm thick, 10 mm in diameter) were obtained from cylindrical bars (ASTM B348, gr5, Titanium Consulting and Trading) by automatic cutting (Brillant 220, ATM GmbH, Mammelzen, Germany, provided with an alumina blade) and employed as substrate in the experimental study. Specimens were manually polished with SiC abrasive papers (120–4,000 grit) and colloidal silica suspension in order to obtain mirror polished Ti6Al4V (Ti6Al4V – MP).

Two different surface modification processes were examined in the present study.

The first one is a chemical treatment aimed at the obtainment of bioactive titanium surfaces for bone integration (Ti6Al4V – CT). It is a patented process which foresees a first etching in hydrofluoric acid followed by controlled oxidation in hydrogen peroxide (Spriano et al., 2007; Ferraris et al., 2011, 2015).

The second one is a coating of titanium boride finalized to better fretting, corrosion and wear resistance (Ti6Al4V – B-coat). The coating is obtained by means of a thermal diffusion process from a boriding powder mixture (50wt% B, 15wt% Na2B4O7, and 35wt% C) in Ar atmosphere for 3.5 h at 800°C, following the protocol of Sarman et al. (2012).

### *In vitro* bioactivity evaluation

In order to evaluate inorganic bioactivity, modified samples (only Ti6Al4V – CT were tested because are intended for bone integration) were soaked in Simulated Body Fluid at pH 7.4 (SBF) (Kokubo and Takadama, 2006) up to 28 days in order to induce apatite precipitation. Refresh of the solution was performed every 2 days. Samples were gently washed at the end of soaking period and dried under a laminar flow cabinet in order to avoid contaminations. In order to compare Ti6Al4V – CT with a surface completely covered by hydroxyapatite, only the sample after 28 days soaking was analyzed in the present research work. Two samples per type were used for *in vitro* bioactivity tests.

### Protein absorption

Bovine Serum Albumin (BSA) (Sigma Aldrich, Saint Louis, USA) was used as model protein for absorption studies. A 20 mg/ml solution of the protein in PBS was prepared in order to mimic the concentration of bovine serum. Each sample (Ti6Al4V – MP, Ti6Al4V – CT, and Ti6Al4V – B-coat) was introduced in a multi well plate and covered with 1ml of BSA solution. The plate was then sealed with parafilm, wrapped in an aluminum foil and stored at 37°C for 2 h in an incubator. At the end of soaking, samples were removed from plates, rinsed in ultrapure water and dried under laminar flow cabinet. Five samples per type were used for protein absorption studies.

### Zeta potential measurements

The zeta potential was measured by means of an electrokinetic analyzer (SurPASS, Anton Paar) equipped with an adjustable gap cell. The surface zeta potential was determined in function of pH in a 0.001 M KCl electrolyte solution varying the solution pH by addition of 0.05M HCl or 0.05M NaOH through the instrument automatic titration unit. Separate couples of samples were used for the acidic and basic titrations in order to avoid artifacts due to surface reactions during the measurement. Four measurements were carried out for each pH point.

### XPS measurements

The surface chemical composition and the presence of specific functional groups were investigated by means of XPS analyses (XPS, XPS, PHI 5000 VERSA PROBE, PHYSICAL ELECTRONICS), in survey and high resolution (C, Ti, O) modes respectively. The high resolution spectra were referenced by setting the hydrocarbon C1s peak to 284.80 eV in order to guarantee the charging effect compensation.

## Results

### Evaluation of the surface modifications

**Figure 2:**
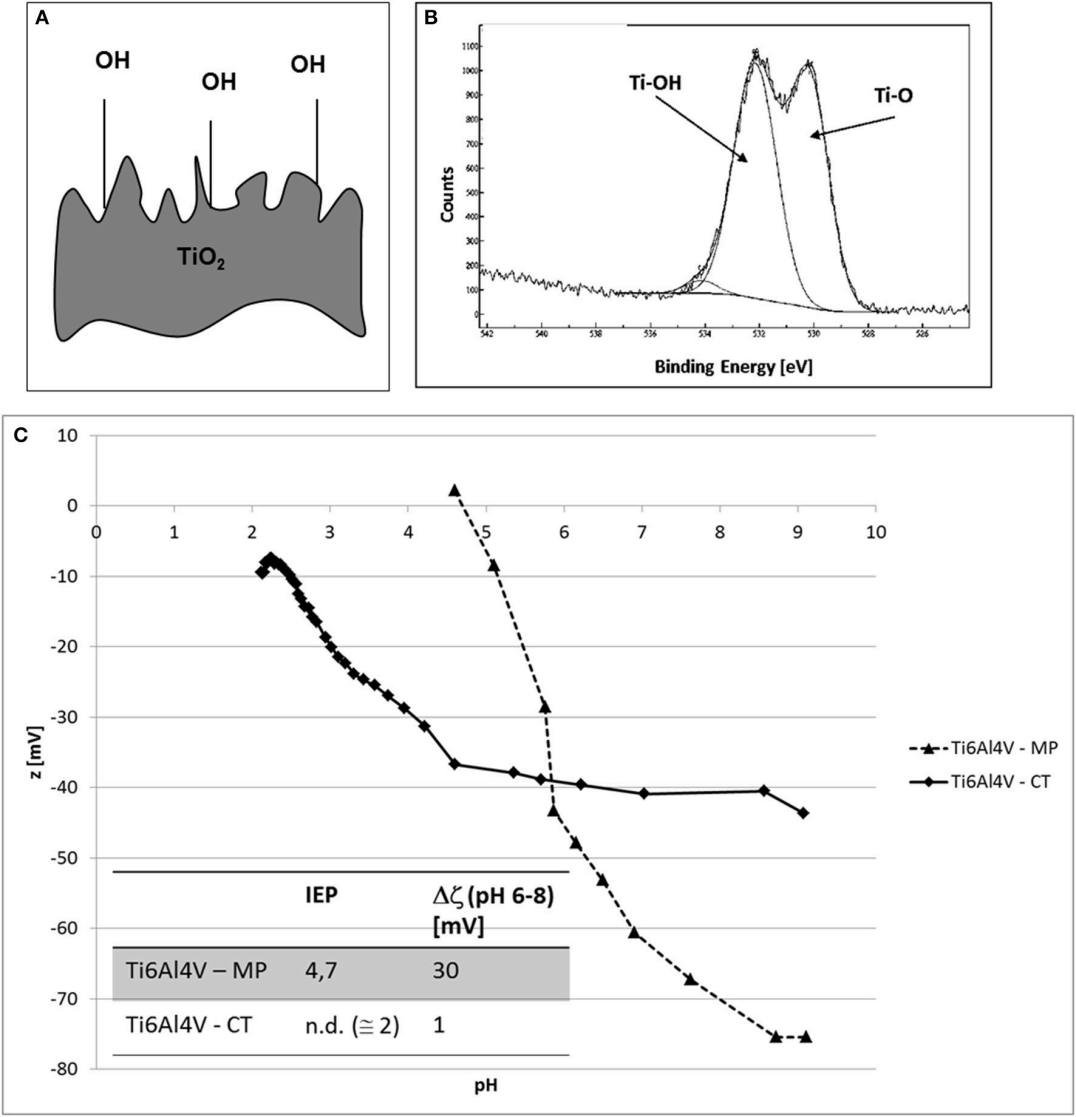
**(A)** Schematic representation of Ti6Al4V – CT surface, **(B)** XPS high resolution spectrum of the oxygen region for Ti6Al4V – CT and **(C)** zeta potential vs. pH measurements of Ti6Al4V – MP and Ti6Al4V – CT samples.

The graphs of zeta potential vs. pH of Ti6Al4V – MP and Ti6Al4V – CT are reported in Figure 2, together with a schematic representation of the surface and its XPS high resolution spectrum of the oxygen region.

The comparison between the two zeta potential titration curves (Figure 2C) highlights an acidic shift of the IEP (from 4.7 to at about 2) after CT treatment. Moreover, a plateau appears in the basic region.

**Figure 3:**
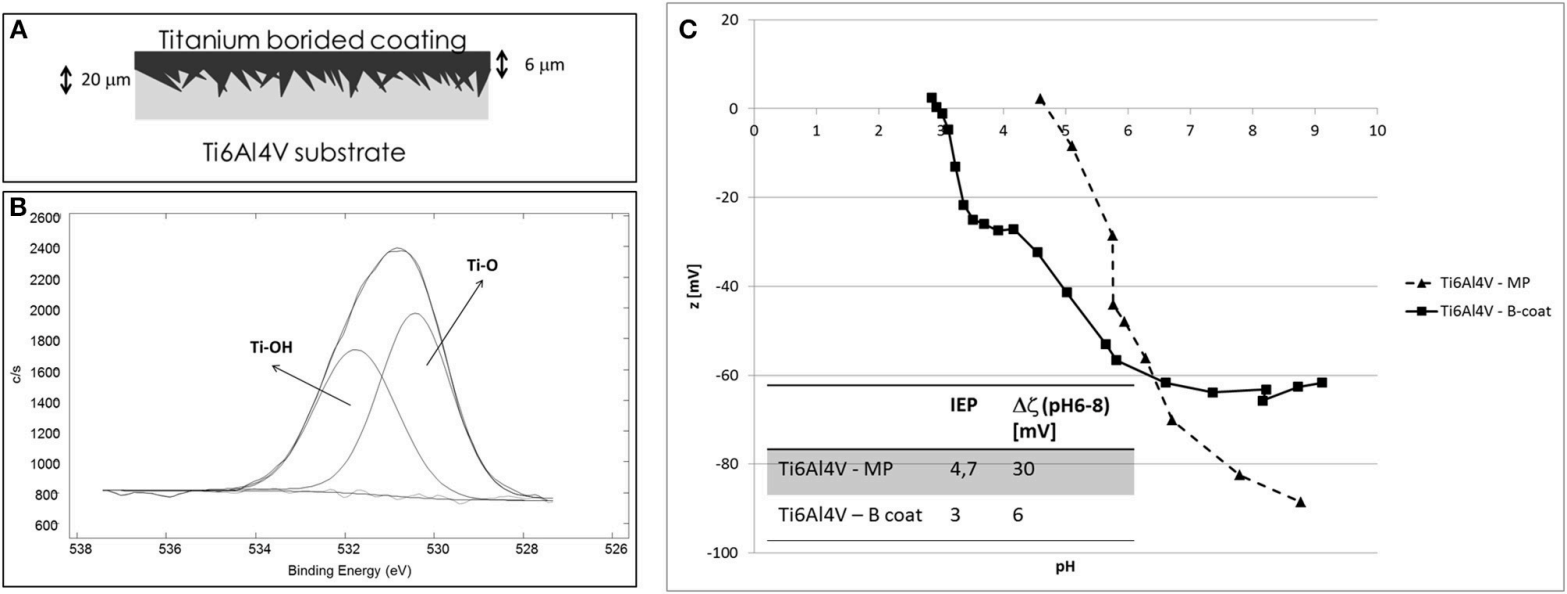
**(A)** Schematic representation of Ti6Al4V – B-coat surface, **(B)** XPS high resolution spectrum of the oxygen region for Ti6Al4V – B-coat, and **(C)** zeta potential vs. pH measurements of Ti6Al4V – MP and Ti6Al4V – B-coat samples.

The graphs of zeta potential vs. pH on Ti6Al4V – MP and Ti6al4V – B-coat are reported in Figure 3 together with a schematic representation of the surface and its XPS high resolution spectrum of the oxygen region.

Similarly to Ti6Al4V – CT sample, the B-coating induces an acidic shift of the IEP (from 4.7 to 3) and the appearance of a plateau in the basic region.

### Investigation of *in vitro* bioactivity

**Figure 4:**
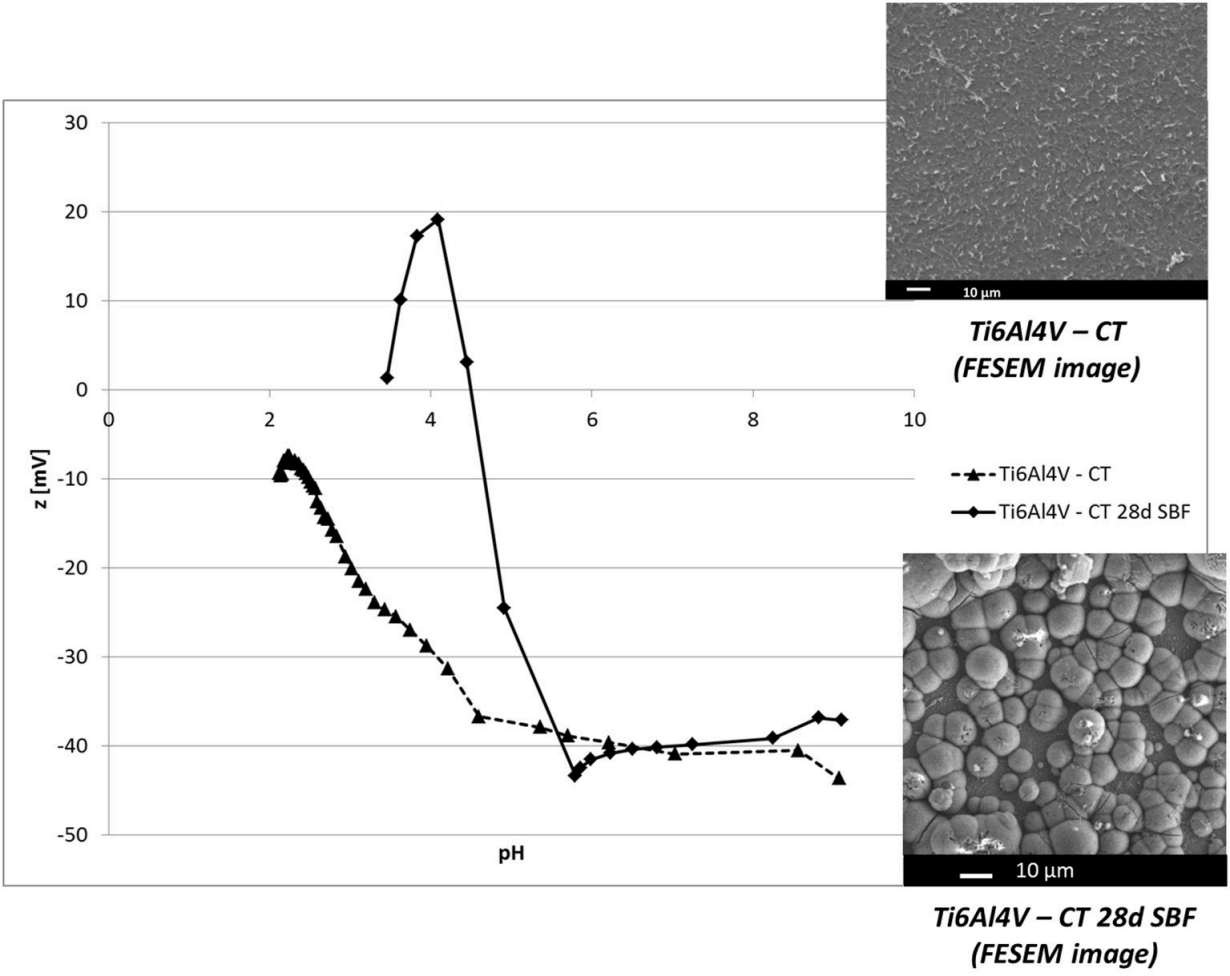
Zeta potential vs. pH of Ti6Al4V – CT and Ti6Al4V – CT after 28 in SBF. Inserts: FESEM image of Ti6Al4V – CT before and after 28 days in SBF (apatite deposition).

The graphs of zeta potential vs. pH of Ti6Al4V – CT and Ti6Al4V – CT after 28 days in SBF are reported in Figure 4, together with the FESEM image of the surface before and after soaking, with a well-developed apatite layer after 28 days in SBF.

The result shows a shift of the IEP of Ti6Al4V – CT surface to more basic values (4.5), close to the ones reported in literature for hydroxyapatite (Botelho et al., 2002).

### Investigation of protein absorption

**Figure 5:**
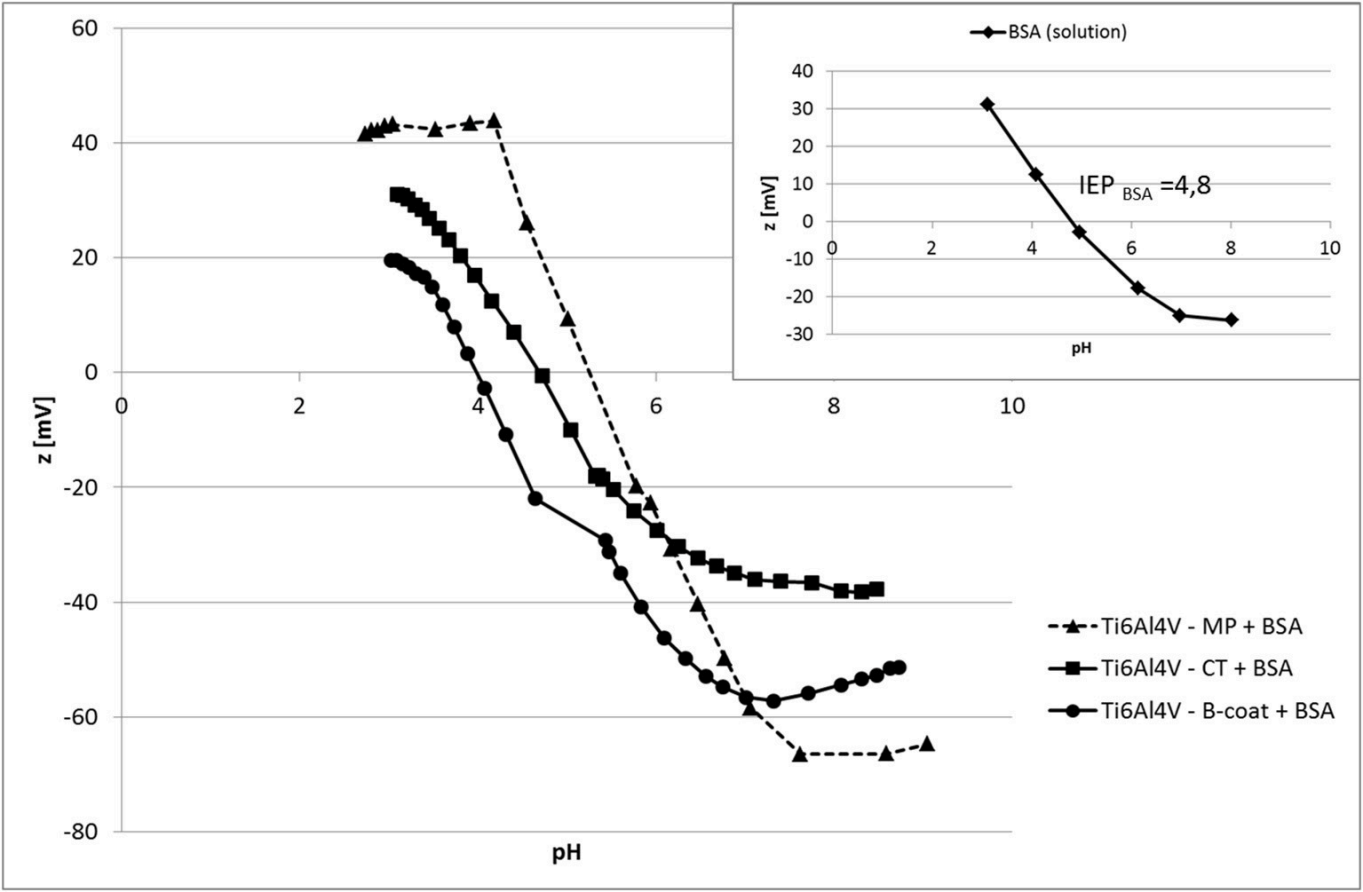
Zeta potential measurements vs. pH of Ti6Al4V – MP, Ti6Al4V – CT, and Ti6Al4V – B-coat after BSA absorption. Insert: zeta potential of an albumin solution (electrophoretic measurements).

Zeta potential measurements vs. pH of Ti6Al4V – MP, Ti6Al4V – CT, and Ti6Al4V – B-coat after BSA absorption are reported in Figure 5, together with the zeta potential titration curve of an albumin solution obtained by electrophoretic measurements (insert).

The IEP of all the tested surface is similar after BSA absorption, even if with some small variations, and close to the one of the BSA solution measured by the electrophoretic technique.

The presence of two plateaus (in the basic and acidic regions) can be noticed for Ti6Al4V – MP, while only the basic plateau is visible on Ti6Al4V – CT and Ti6Al4V – B-coat samples.

## Discussion

Zeta potential measurements (based on the electrophoretic mobility of particles) are widely employed for the characterization of colloidal suspensions (Bhattacharjee, 2016). On the other hand, zeta potential measurements on solid surfaces are less usual. Contact angle titatration has been proposed for the evaluation of the isoelectric point of solid samples (Chedov and Logan, 2004), but the result is limited to the IEP value and measurements are quite complex with consequent difficulties in the obtainment of reliable results (Spriano et al., 2017). Electrokinetic measurements of the zeta potential of solid surfaces can be more interesting for the determination of both the IEP value and also of the zeta potential in function of pH, making possible a more in depth understanding of surface properties (Cai et al., 2006; Luxbacher, 2014; Spriano et al., 2017).

Characterization of different surfaces by means of the streaming potential technique for the determination of zeta potential of solid surfaces can give interesting and useful information about surface chemistry, as here discussed. The IEP of Ti6Al4V – MP sample is 4.7, according to the values reported in literature for titanium surfaces (Bal and Rahaman, 2012). For both the considered treatments (Ti6Al4V – CT and Ti6Al4V – B-coat), a shift of the IEP to more acidic values, compared to bare Ti6Al4V, can be noticed, with major evidence in the case of Ti6Al4V – CT. In addition, the appearance of a plateau in the basic region can be noticed. These two results can be associated with the surface enrichment in hydroxyl groups with acidic behavior after the surface modification process, in accordance with XPS results (Figures 1B, 2B). The plateau starts around pH 4.5 in the case of Ti6Al4V – CT and around pH6 in the case of Ti6Al4V – B-coat: this evidence can be related to a different acidic strength of the OH groups on the two different surfaces. They act as a much more strong acid in the first case with a complete deprotonation at a lower pH value. The slope of the curve before the plateau can give information about surface hydrophilicity/hydrophobicity, in fact a hydrophobic surface, which weakly bonds water molecules and easily exchange them with ions from the solution, shows an high slope. In fact, Ti6Al4V – MP shows wettability lower than Ti6Al4V – CT, in accordance with the wettability measurements performed by the sessile drop method using ultrapure water as wetting fluid (81.4° for Ti6Al4V – MP and 76.0° for Ti6Al4V – CT, as reported in Ferraris et al., 2011). Both hydrophilicity/hydrophobicity and acidic/basic behavior of surface functional groups have a great effect on bioactivity and surface reactions of a bio-surface with physiological fluid, that is why this kind of measurement is of great relevance.

After SBF soaking for 28 days, the surface of Ti6Al4V – CT is completely covered by a hydroxyapatite layer (FESEM observation in the insert of Figure 4). Apatite deposition on Ti6Al4V – CT surfaces was demonstrated by the authors starting from 14 days soaking (Ferraris et al., 2011).

Zeta potential measurements in function of pH revealed a significant change on the surface, in fact the IEP results shifted close to the one of apatite (Botelho et al., 2002) and the shape of the curve changes, as indication of a different surface chemistry. A plateau persists in the basic region in accordance with the high density of acidic OH groups in hydroxyapatite. In the acid range, chemical stability of HA can be estimated by the maximum at pH 4, at this pH in fact the z value becomes unstable, that is related to its reaction with the solution: this information can be related to “maturation” and chemical stability of the apatite layer. All things considered, zeta potential titration curve can be an interesting tool in order to characterize the mechanism, kinetic and type of HA formed on bioactive materials.

Zeta potential measurement can be also a technique for evaluate protein absorption on bio-surfaces. The IEP moves in the tested materials close to the one measured with pure albumin by electrophoretic technique (Figure 5, insert) confirming the effective absorption of BSA on all the tested materials. The shape of the curves, however, is different among the tested surfaces. In fact, while two plateau (one in the basic region and one in the acidic one) are observable on Ti6Al4V – MP +BSA, there is only the acidic one on Ti6Al4V – CT + BSA and Ti6Al4V – B-coat+BSA. The acidic plateau can be associated with the presence of basic functional groups (NH_2_ groups of the BSA molecule), while the basic one with acidic functional groups (COOH of BSA molecule). The here reported results suggest a different disposition of BSA on Ti6Al4V – MP compared to Ti6Al4V – CT and Ti6Al4V – B-coat. This difference can be associated with the presence of OH groups on both Ti6Al4V – CT and Ti6Al4V – B-coat, as underlined by XPS and zeta potential measurements, previously discussed, while the Ti6Al4V – MP surface is a hydrophobic surface without specific functional groups. Moreover, the slope of the curve before the plateau is higher for Ti6Al4V – MP than for Ti6Al4V – CT and Ti6Al4V – B-coat, suggesting that BSA is adsorbed in a highly hydrophilic configuration onto Ti6Al4V – CT and Ti6Al4V – B-coat surfaces. The amount and configuration of protein adsorption on bio-surfaces is of great relevance in determining their biological response and zeta potential titration curves can give a contribution to understand these issues.

In conclusion, zeta potential titration curves of bulk biomaterials, linked to XPS, wettability and other chemical surface analyses, can be of great interest in order to understand the behavior of bio-surfaces in contact with SBF or protein solutions, to design novel biomaterials and to explain their *in vivo* behavior. The technique is extremely sensitive and, in combination with other measurements (e.g., XPS) can support an in depth understanding of surface modifications after chemical treatments or interface reactions with fluids of biological interest. In the present study the possibility to detect changes in the isoelectric point of the surface after chemical treatments, apatite precipitation or protein absorption has been demonstrated for the first time on solid titanium surfaces. Moreover it was observed that the technique is suitable for the investigation of surface functional groups exposed on the surfaces in different media and at different pH. In order to identify these groups the combination of zeta potential measurements and XPS analyses result crucial.

## Author contributions

SF, MC, and VP: performed the experiments, discussed the results and wrote the first draft of the work; BS: participate to experimental activity, discussion, and paper drafting; SS: coordinated the work, discussed the results, participated to the first draft of the paper, and performed its final revision.

### Conflict of interest statement

The authors declare that the research was conducted in the absence of any commercial or financial relationships that could be construed as a potential conflict of interest.
